# Vinorelbine in bladder-preserving multimodality treatment for muscle-invasive bladder cancer—a valid option for cisplatin-unfit patients?

**DOI:** 10.1007/s00066-021-01837-7

**Published:** 2021-08-19

**Authors:** C. R. Arnold, A. K. Lindner, G. Schachtner, G. Tulchiner, N. Tulchiner, J. Mangesius, M. Maffei, W. Horninger, O. Kouvaiou, P. Lukas, U. Ganswindt, R. Pichler, S. Skvortsov

**Affiliations:** 1 Department of Therapeutic Radiology and Oncology, Medical University of Innsbruck, Anichstraße 35, 6020 Innsbruck, Austria; 2 Department of Urology, Medical University of Innsbruck, Anichstraße 35, 6020 Innsbruck, Austria; 3 Department of Radiation Oncology, General Hospital Bolzano, Lorenz Böhler Straße 5, 39100 Bolzano, Italy

**Keywords:** MIBC, BPMT, Chemoradiotherapy, Bladder cancer, Organ preservation

## Abstract

**Purpose:**

Treatment of muscle-invasive bladder cancer (MIBC) remains challenging, especially for elderly and/or comorbid patients. Patients who are unfit for or refuse surgery should receive bladder-preserving multimodality treatment (BPMT), consisting of transurethral resection of the bladder tumor (TURB) followed by combined chemoradiotherapy (CRT). We aimed to investigate the effectiveness of vinorelbine, a chemotherapeutic agent not routinely used for MIBC, in patients referred to CRT who are unfit for standard chemotherapy and would thus rely solely on radiotherapy (RT).

**Methods:**

We retrospectively analyzed 52 consecutive patients with MIBC who received standard CRT with cisplatin (*n* = 14), CRT with vinorelbine (*n* = 26), or RT alone (*n* = 12). Primary endpoints were median overall survival (OS) and median cancer-specific survival (CSS). Secondary endpoints were median local control (LC), median distant control (DC), and OS, CSS, LC, and DC after 1, 2, and 3 years, respectively.

**Results:**

Median OS and CSS were significantly higher for patients who received vinorelbine as compared to RT alone (OS 8 vs. 22 months, *p* = 0.003; CSS 11 months vs. not reached, *p* = 0.001). Median LC and DC did not differ significantly between groups. Vinorelbine was well tolerated with no reported side effects >grade II.

**Conclusion:**

Our results suggest that CRT with vinorelbine is well tolerated and superior to RT alone in terms of OS and CSS. Therefore, this treatment regime might constitute a new treatment option for patients with MIBC who are unfit for or refuse surgery or standard chemotherapy. This study encourages a randomized controlled trial to compare this new regime to current standard therapies.

## Introduction

Muscle-invasive bladder cancer (MIBC) is associated with high morbidity and mortality rates if not treated optimally [1]. Hence, prompt and adequate treatment is of vital importance. According to current European Association of Urology (EAU) guidelines, there are two treatment options for localized MIBC: radical cystectomy (RC) as the current gold standard and bladder-preserving multimodality treatment (BPMT) as a valid alternative for selected, well-informed, and compliant patients, especially when RC is not an option. BPMT consists of maximal transurethral resection of the bladder tumor (TURB) followed by chemoradiotherapy (CRT) [2]. Survival outcomes after BPMT for carefully selected patients are comparable to RC but without the risks of perioperative mortality and morbidity [3–6]. For BPMT, patients usually receive cisplatin or mitomycin C plus fluorouracil as radiosensitizer to potentiate radiation therapy (RT), resulting in 5‑year cancer-specific survival and overall survival rates from 50 to 82% and from 36 to 74%, respectively [7]. However, nearly half of the patients undergoing RC are cisplatin-ineligible based on poor renal function, various comorbidities, or older age at first diagnosis [8, 9]. These patients then rely solely on RT, which is known to be significantly inferior to combined CRT [10–14]. Improving treatment options for this group of selected patients is challenging but paramount.

Vinorelbine is a semi-synthetic vinca alkaloid that binds to tubulin and inhibits microtubule polymerization during mitosis. Vinorelbine can be given intravenously or orally and is routinely used in combination with cisplatin as first-line therapy in the definitive or adjuvant treatment of NSCLC [15, 16]. Additionally, vinorelbine monotherapy can be considered in previously treated patients and elderly or unfit patients with advanced NSCLC. Compared to other chemotherapies, especially platin derivates, vinorelbine is considerably better tolerated, with the most common toxicities being neutropenia and gastrointestinal side effects [17]. However, aside from a case report in recurrent small-cell bladder cancer [18], there are, to our knowledge, currently no reports on vinorelbine in MIBC.

## Materials and methods

### Inclusion and exclusion criteria

For this retrospective analysis, we included all patients with histologically confirmed localized MIBC for whom RC was contraindicated due to comorbidities or due to patient preference and who underwent primary BPMT at the Department of Therapeutic Radiology and Oncology, Medical University of Innsbruck, or the Department of Radiation Oncology, General Hospital Bolzano, between 07/2008 and 12/2018. BPMT consisted of maximal TURB and CRT with cisplatin. Patients that were unfit for cisplatin were offered vinorelbine as radiosensitizer as an institutional practice. If patients refused or were unfit for vinorelbine, they were treated by RT alone. Thus, patients were stratified into three treatment groups: i) CRT with cisplatin, ii) CRT with vinorelbine, and iii) RT alone. We excluded patients with non-pure urothelial cancer and those with evidence of distant or local metastases on pretherapeutic standard staging imaging. Patient data were extracted from our medical and radiation records. The study was approved by the local ethics committee (study number 1270/2018) and was conducted in accordance with the 1964 Helsinki declaration and its later amendments or comparable ethical standards [19].

### Multimodality therapy

Maximal TURB was performed 4 to 6 weeks (median 5 weeks) prior to RT. Subsequently, all patients received 3D conformal RT with daily fractions of 1.8 Gy on 5 consecutive days to a total dose of 50.4 Gy to the pelvic lymphatics and 59.4 Gy to the whole bladder. RT was performed in a supine position with an emptied bladder. First choice for concomitant systemic therapy was cisplatin at a dose of 25 mg/m^2^ on days 1 to 5 and 29 to 33. Cisplatin-unfit patients were defined by the presence of at least one of the following criteria: WHO PS of 2 or higher, impaired renal function (GFR < 60 ml/min), audiometric hearing loss defined by the Common Terminology Criteria for Adverse Events (CTCAE) as grade II or higher, peripheral neuropathy grade II or higher (CTCAE), or heart failure NYHA class III or higher [20, 21]. Cisplatin-unfit patients or those who refused cisplatin received the radiosensitizer vinorelbine at a dose of 40 mg/m^2^ (orally, *n* = 26) or 15 mg/m^2^ (intravenously, *n* = 3) once a week during RT. If patients refused or were unfit for vinorelbine as well, they received RT alone.

### Endpoints

Primary endpoints were median overall survival (OS) and median cancer-specific survival (CSS). Secondary endpoints were median local control (LC), median distant control (DC), and OS, CSS, LC, and DC after 1, 2, and 3 years, respectively.

### Follow-up protocols, assessment of response, and treatment of recurrence

Three months after completion of RT, first cystoscopy with urine cytology (bladder washings and voided urine) was performed and response to BPMT was assessed. Complete response (CR) to therapy was defined as no visible tumor on cystoscopy, negative urine cytology, and a negative TURB. If no cystoscopy post treatment was performed, response status was assessed using CT or MR imaging. Follow-up visits were scheduled every 3 months in the first 2 years, then at 6‑monthly intervals until the end of the fifth year, and once a year thereafter [2, 22]. Each follow-up visit included cystoscopy, voided urine and bladder washing cytology, standard imaging (i.e., contrast-enhanced chest and abdominopelvic CT scan every 6 months in the first 2 years, then once a year thereafter or immediately in the case of suspected MIBC recurrence). Re-TURB was performed in case of local tumor recurrence or persistence. In case of muscle-invasive recurrence after BPMT, patients were recommended to undergo salvage cystectomy. Those with non-muscle-invasive recurrence after BPMT were treated with TURB and adjuvant intravesical therapy based on tumor staging and grading according to the EAU guidelines [23]. Patients with systemic progression were recommended to receive palliative systemic chemotherapy and/or optionally RT in case of single or symptomatic metastases. If further treatment was refused by the patient, best supportive care was initiated.

### Statistical analysis

LC, DC, OS, and CSS were defined as the timespan from the date of diagnosis to the detection of local recurrence, detection of distant recurrence, death from any cause, or cancer-related death, respectively. In the case of no respective event, patients were censored at the date of last control. Survival rates and curves were calculated using the Kaplan–Meier product-limit estimation approach. Stratified survival curves were compared using the log-rank test. *P*-values below 0.05 were considered as statistically significant. Statistical analyses were performed using SPSS version 26 (IBM Corp., Armonk, NY, USA).

## Results

### Patient characteristics

In total, 52 patients with a mean age of 77.5 years (median 80, range 48–91 years) were included in this study. Twelve patients (23.1%) were female. Median Karnofsky performance index (KPI) was 8 (range 6–10). According to treatment, patients were stratified in three groups: i) CRT with cisplatin (*n* = 14, 26.9%), ii) CRT with vinorelbine (*n* = 26, 50.0%), and iii) RT alone (*n* = 12, 23.1%). Hydronephrosis was present at diagnosis in 19 patients (36.5%). Of those, 3 patients (15.8%) received sole RT, 12 patients (63.2%) were treated with CRT with vinorelbine, and 4 patients (21.0%) received CRT with cisplatin. Creatinine clearance ranged from 17.9 to 125.9 ml/min (mean 58.5 ml/min ± 25.4). The reason for not having received primary RC was medical unfitness in 34 patients (65.4%) and refusal by the patient in 15 cases (28.8%). The reason was unknown in 3 cases (5.8%). Of all patients, 4 (7.7%) had positive lymph nodes on pretherapeutic imaging. Patient characteristics are summarized in Table 1.

**Table 1:** Patient characteristics

	All patients	RT only	CRT with vinorelbine	CRT with cisplatin
*n*	52	12	26	14
*Male, n (%)*	40 (76.9)	7 (58.3)	21 (80.8)	12 (85.7)
*Age (years), median (range)*	80 (48–91)	84 (69–88)	80.5 (54–91)	72.5 (48–86)
*Age (years), mean (±* *SD)*	77.5 (± 9.4)	81.8 (± 6.1)	78.4 (± 9.3)	72.1 (± 9.8)
*KPI, median (range)*	8.0 (6–10)	7.5 (6–9)	8.5 (6–9)	9.0 (7–10)
*KPI, mean (±* *SD)*	8.2 (± 1.02)	7.7 (± 1.0)	8.2 (± 1.0)	8.5 (± 1.1)
*Lymph node status at Dg., n (%)*	4 (7.7)	0 (0.0)	3 (11.5)	1 (7.1)
*Creatine clearance at Dg. (ml/min), mean (±* *SD)*	58.5 (± 25.4)	44.6 (± 20.9)	50.4 (± 17.5)	85.6 (± 21.5)
*Hydronephrosis at Dg., n (%)*	19 (36.5)	3 (25.0)	12 (46.2)	4 (28.6)
*Reason for no RC, n (%)*				
Unfit	34 (65.4)	8 (66.7)	20 (76.9)	6 (42.9)
Refused	15 (28.8)	3 (25.0)	6 (23.1)	6 (42.9)
Unknown	3 (5.8)	1 (8.3)	0 (0.0)	2 (14.3)

*Dg.* diagnosis, *RC* radical cystectomy, *RT* radiation therapy, *CRT* chemoradiotherapy, *KPI* Karnofsky performance index

### Response to BPMT and patterns of failure

In total, 36 patients (69.2%) achieved a complete remission, 2 patients (3.9%) showed a partial remission, 1 patient (1.9%) had stable disease, and 7 patients (13.5%) displayed progressive disease. Six patients (11.5%) had no posttherapeutic cystoscopy or CT scan. Hence, response to BPMT could not be assessed. There was no difference in response to BPMT between the three treatment groups. After a median follow-up of 25 months (range 4–91 months), 12 patients (23.1%) developed a local recurrence (9 of which were muscle-invasive), while 8 patients (15.4%) suffered from distant recurrence, and 6 patients (11.5%) had local as well as distant recurrences. One patient (1.9%) with isolated muscle-invasive local recurrence underwent salvage cystectomy, while the other patients with muscle-invasive local recurrence were assigned to best supportive care. Of the three patients with non-muscle-invasive local recurrence, two received TURB followed by instillation therapy and one was treated with TURB alone. Patients with distant metastases (± local recurrences) underwent systemic chemotherapy, local RT, or best supportive care, depending on the extent of disease. Response to BPMT and patterns of failure are summarized in Table 2.

**Table 2:** Response to therapy and patterns of failure

	All patients	RT only	CRT with vinorelbine	CRT with cisplatin
*Response to BPMT, n (%)*				
CR	36 (69.2)	7 (58.3)	18 (69.2)	11 (78.6)
No CR	10 (19.2)	3 (25.0)	6 (23.1)	1 (7.1)
n/a	6 (11.5)	2 (16.7)	2 (7.7)	2 (14.3)
*Recurrence*				
None	26 (50.0)	5 (41.7)	14 (53.8)	7 (50.0)
Local	12 (23.1)	5 (41.7)	5 (19.2)	2 (14.3)
Distant	8 (15.4)	1 (8.3)	3 (11.5)	4 (28.6)
Local and distant	6 (11.5)	1 (8.3)	4 (15.4)	1 (7.1)

*RT* radiation therapy, *CRT* chemoradiotherapy, *BPMT* bladder-preserving multimodal therapy, *CR* complete response

### Survival

**Fig. 1:**
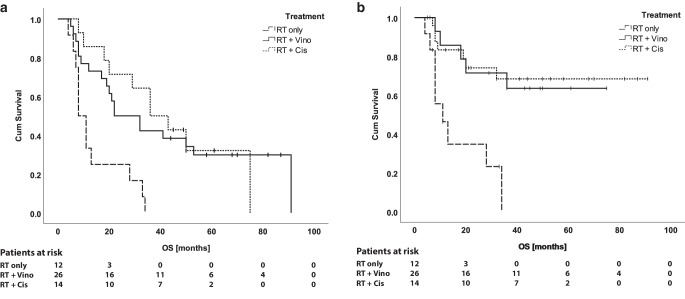
Overall survival (**a**) and cancer-specific survival (**b**) curves estimated with the Kaplan–Meier method stratified by treatment

Median OS of all patients was 22 months (range 4–91 months). Median OS for patients who received cisplatin, vinorelbine, or RT only was 36, 22, and 8 months, respectively. There was a statistically significant difference in median OS between vinorelbine and RT only (*p* = 0.003, Fig. 1a). One-, 2‑, and 3‑year survival for all patients was 69.2%, 50.0%, and 38.5%, respectively. One-, 2‑, and 3‑year survival for patients who received cisplatin, vinorelbine, or RT only was 85.7%, 71.4%, and 64.3% (cisplatin), 76.9%, 50.0%, and 42.3% (vinorelbine), and 33.3%, 25.0%, and 0.0% (RT only), respectively. Median OS for patients with local recurrence (*n* = 12) was 28 months. Interestingly, the 3 patients with non-muscle-invasive local recurrence reached an OS of 45, 61, and 82 months, respectively.

Median CSS for all patients was 32 months. Median CSS for patients who received cisplatin or RT only was 36 and 11 months, respectively. Median CSS for patients who received vinorelbine was not reached. Again, there was a significant difference in CSS in log-rank test between vinorelbine and RT only (*p* = 0.001, Fig. 1b). One-, 2‑, and 3‑year CSS was 75.0%, 59.6%, and 53.8% (all patients), 85.7%, 71.4%, and 71.4% (cisplatin), 84.6%, 65.4%, and 61.5% (vinorelbine), and 41.7%, 33.3%, and 16.7% (RT only), respectively. Survival data are summarized in Table 3.

**Table 3:** Survival data

	All patients	RT only	CRT with vinorelbine	CRT with cisplatin	RT only vs. CRT vinorelbine
*Median OS (months)*	22	8	22	36	*p* = 0.003
1a-OS (%)	69.2	33.3	76.9	85.7	
2a-OS (%)	50.0	25.0	50.0	71.4	
3a-OS (%)	38.5	0.0	42.3	64.3	
*Median CSS (months)*	32	11	n. r.	36	*p* = 0.001
1a-CSS (%)	75.0	41.7	84.6	85.7	
2a-CSS (%)	59.6	33.3	65.4	71.4	
3a-CSS (%)	53.8	16.7	61.5	71.4	

A *p*-value below 0.05 was considered statistically significant. Comparisons were performed with the log-rank test

*OS* overall survival, *CSS* cancer specific survival, *RT* radiation therapy, *CRT* chemoradiotherapy, *n.* *r.* not reached, *1a* one-year, *2a* two-year, *3a* three-year

### Local and distant control rates

Median LC and median DC for all patients as well as for each treatment group were not reached. One-, 2‑, and 3‑year LC was 82.7%, 73.1%, and 67.3% (all patients), 100.0%, 78.6%, and 78.6% (cisplatin), 80.8%, 67.9%, and 69.2% (vinorelbine), and 66.7%, 58.3%, and 50.0% (RT only), respectively. One-, 2‑, and 3‑year DC was 86.5%, 76.9%, and 75.0% (all patients), 78.6%, 71.4%, and 71.4% (cisplatin), 92.3%, 76.9%, and 73.1% (vinorelbine), and 83.3%, 83.3%, and 83.3% (RT only), respectively. There was no statistically significant difference in median LC or median DC between vinorelbine and RT only. Local and distant control data are summarized in Table 4.

**Table 4:** Local and distant control data

	All patients	RT only	CRT with vinorelbine	CRT with cisplatin	RT only vs. CRT vinorelbine
*Median LC (months)*	n. r.	n. r.	n. r.	n. r.	n. s.
1a-LC (%)	82.7	66.7	80.8	100	
2a-LC (%)	73.1	58.3	76.9	78.6	
3a-LC (%)	67.3	50.0	69.2	78.6	
*Median DC (months)*	n. r.	n. r.	n. r.	n. r.	n. s.
1a-DC (%)	86.5	83.3	92.3	78.6	
2a-DC (%)	76.9	83.3	76.9	71.4	
3a-DC (%)	75.0	83.3	73.1	71.4	

A *p*-value below 0.05 was considered statistically significant. Comparisons were performed with the log-rank test

*LC* local control, *DC* distant control, *n.* *r*. not reached, *n.* *s.* not significant, *RT* radiation therapy, *CRT* chemoradiotherapy, *1a* one-year, *2a* two-year, *3a* three-year

### Side effects

Neither radiation nor vinorelbine nor cisplatin caused side effects >grade II. Minor side effects induced by radiation were an increased frequency of micturition and/or defecation, dysuria, diarrhea, and radiodermatitis, none of which required therapeutic intervention. Vinorelbine was well tolerated with no patient reporting gastrointestinal adverse effects, loss of hair, fatigue, or neuropathy. Regular blood work did not show clinically significant hematological side effects.

## Discussion

In this retrospective study we present the results of 52 patients with MIBC who were treated in an organ-preserving intention with multimodal treatment using CRT with two different radiosensitizing agents (cisplatin and vinorelbine) or sole RT.

The most important finding of this study is certainly that using vinorelbine as a radiosensitizer improved survival in patients who were unfit for cisplatin compared to RT alone. While vinorelbine is part of several treatment schemes for patients with NSCLC [15, 16], this is, to our knowledge, the first report of vinorelbine in the treatment of MIBC. This is of special importance, as this cancer is commonly found in elderly and/or multimorbid patients who are often unfit for or refuse highly toxic cisplatin-based chemotherapies [24, 25]. There are few alternatives to cisplatin for this particular patient population. Data exist for the combination of fluorouracil and mitomycin C from one large randomized phase 3 trial with 360 patients [10], or gemcitabine, which has been tested in a randomized phase 2 trial with 66 patients [26]. Importantly, both treatment regimens are accompanied by significant toxicities, albeit both being usually better-tolerated chemotherapeutics as compared to cisplatin. In the prior study, 36% of patients in the fluorouracil/mitomycin C group displayed any CTCAE grade 3–5 toxic effects. In the second study, 55% of patients who received gemcitabine suffered from any grade 3–4 toxicities and 42% from grade 3–4 hematological toxicities. In contrast, there are reports on the high tolerability of vinorelbine, especially in frail and elderly patients [27, 28]. These data are supported by the findings of our study, which showed no grade 3–4 toxicities in the vinorelbine group. Additionally, fluorouracil/mitomycin C and gemcitabine both implicate the necessity of intravenous administration of the substances. Vinorelbine, in contrast, can be administered orally. This increases treatment convenience and, in further consequence, patient compliance, which must not be underestimated in elderly and/or frail patients [29]. Finally, if patients do not qualify for or refuse any of these standard intravenous chemotherapy schemes, they would then rely solely on RT. Taken together, vinorelbine offers a new therapeutic approach for this group of selected patients.

In our cohort, vinorelbine was able to significantly improve both median OS as well as median CSS compared to RT alone (Fig. 1). In line with this, vinorelbine also improved 1‑, 2‑, and 3‑year OS and CSS (Table 3). This clearly indicates a survival benefit for patients who were unfit for or refused standard chemotherapies and would therefore be treated by the sole means of RT, which is known to be inferior compared to combined CRT [10–14]. Unsurprisingly, the best OS and CSS was seen in the cisplatin group. Compared to sole irradiation, vinorelbine, similarly to cisplatin, also improved 1‑, 2‑, and 3‑year local control, emphasizing its role as a radiosensitizing agent (Table 4; [30]). In contrast, 1‑, 2‑, or 3‑year distant control did not differ between the three groups (Table 4). This might be caused by the generally lower systemic activity of a chemotherapeutic agent when used as radiosensitizer as compared to a definitive chemotherapy where the cumulative dose is substantially higher (e.g., cumulative dose of cisplatin in this study 200 mg/m^2^ vs. cumulative dose of cisplatin in the definitive treatment of esophageal cancer 600 mg/m^2^).

Importantly, vinorelbine is known to be well tolerated [17, 27]. This was confirmed in our study, as we observed no clinically significant vinorelbine-associated side effects. There were also no radiation-induced adverse reactions ≥grade II in any group, making this treatment highly tolerable. This is of special importance, as these patients are often frail and/or elderly or deliberately refuse toxic chemotherapies for fear of side effects.

Compared to other studies, overall survival results in our study were relatively low [31, 32]. There are several explanations for this finding. First, two thirds of patients were medically unfit for cystectomy, indicating a generally compromised condition of these patients. Second, three quarters of the patients did not receive cisplatin. This resulted in most patients being suboptimal patients for curative bladder-preserving therapy [33]. This is reflected by the fact that survival for patients who did receive cisplatin was significantly better. Third, the median age of the patients in our study was 80 years, which is considerably higher than in most other studies [31, 32]. Fourth, one third of patients presented with hydronephrosis at diagnosis, which is known to be a negative prognostic factor for patients with MIBC [34]. Fifth, one fourth of patients did not receive any chemotherapy at all, which is known to be less effective than concomitant CRT [10–13]. Taken together, a large proportion of our patients were treated in a palliative intention and direct comparison to studies with curative treatment is inappropriate. Nevertheless, treatment for patients in a palliative setting needs to be optimized and vinorelbine might be effective in this situation.

The dispute is ongoing regarding whether organ-sparing BPMT is an acceptable alternative to RC for patients with MIBC. Randomized controlled trials are lacking, but current evidence suggests that for appropriately selected patients, BPMT can yield survival outcomes similar to those of patients who undergo RC [35–37]. In our study, survival of patients treated with CRT with cisplatin is relatively low compared to other studies [38], likely due to the aforementioned reasons. Therefore, our results need to be interpreted carefully when comparing them to other studies of BPMT or RC.

Certainly, this study has limitations. First, this is a retrospective analysis with all its inherent disadvantages, such as missing randomization. When comparing baseline characteristics, we found that patients in the cisplatin group were younger and had better creatinine clearance than patients in the other groups. This is not surprising considering that old age and compromised renal function are often exclusion criteria from platin-based chemotherapy [39]. Importantly, we could not detect significant differences in age, KPI, and creatinine clearance between the vinorelbine group and the RT-only group, even though patients in the vinorelbine group tended to be younger and have a higher KPI. On the other hand, 12% of patients in the vinorelbine group (versus none in the RT-only group) had positive lymph nodes on pretreatment imaging, probably compromising the benefits of lower age and higher KPI. Second, we did not analyze patients who received fluorouracil/mitomycin C. The respective study was published in 2012 [10] and the number of patients treated with this regime during the inclusion period of our study was too low. Certainly, it would be highly interesting to compare efficacy data of CRT with cisplatin, fluorouracil/mitomycin C, and vinorelbine. Third, group size, especially in the cisplatin and RT-only groups, was relatively small, demanding careful interpretation of our results. While no definitive conclusions can be drawn from our results, they at least encourage the initiation of follow-up studies, especially of a randomized controlled trial, to further investigate this interesting new approach.

In conclusion, the results of this retrospective analysis suggest that vinorelbine in combination with RT is superior to RT alone in terms of overall and cancer-specific survival in patients who are unfit for or refuse RC and/or CRT with cisplatin. Additionally, this combination is exceptionally well tolerated, making it an interesting treatment strategy for selected frail and/or elderly patients with MIBC in curative intent. In order to validate our findings and strengthen the evidence for this new therapeutic option, further investigation with larger randomized trials is encouraged.
